# Zebrafish transposable elements show extensive diversification in age, genomic distribution, and developmental expression

**DOI:** 10.1101/gr.275655.121

**Published:** 2022-07

**Authors:** Ni-Chen Chang, Quirze Rovira, Jonathan Wells, Cédric Feschotte, Juan M. Vaquerizas

**Affiliations:** 1 Department of Molecular Biology and Genetics, Cornell University, Ithaca, New York 14850, USA;; 2 Max Planck Institute for Molecular Biomedicine, 48149 Muenster, Germany;; 3 MRC London Institute of Medical Sciences, London W12 0NN, United Kingdom;; 4 Institute of Clinical Sciences, Faculty of Medicine, Imperial College London, London W12 0NN, United Kingdom

## Abstract

There is considerable interest in understanding the effect of transposable elements (TEs) on embryonic development. Studies in humans and mice are limited by the difficulty of working with mammalian embryos and by the relative scarcity of active TEs in these organisms. The zebrafish is an outstanding model for the study of vertebrate development, and over half of its genome consists of diverse TEs. However, zebrafish TEs remain poorly characterized. Here we describe the demography and genomic distribution of zebrafish TEs and their expression throughout embryogenesis using bulk and single-cell RNA sequencing data. These results reveal a highly dynamic genomic ecosystem comprising nearly 2000 distinct TE families, which vary in copy number by four orders of magnitude and span a wide range of ages. Longer retroelements tend to be retained in intergenic regions, whereas short interspersed nuclear elements (SINEs) and DNA transposons are more frequently found nearby or within genes. Locus-specific mapping of TE expression reveals extensive TE transcription during development. Although two-thirds of TE transcripts are likely driven by nearby gene promoters, we still observe stage- and tissue-specific expression patterns in self-regulated TEs. Long terminal repeat (LTR) retroelements are most transcriptionally active immediately following zygotic genome activation, whereas DNA transposons are enriched among transcripts expressed in later stages of development. Single-cell analysis reveals several endogenous retroviruses expressed in specific somatic cell lineages. Overall, our study provides a valuable resource for using zebrafish as a model to study the impact of TEs on vertebrate development.

Transposable elements (TEs) are selfish genetic elements that replicate and mobilize within host genomes. They have colonized all vertebrate species sequenced to date but with differential success, accounting for between 4% and 60% of their genomes (Sotero-Caio et al. 2017). The success of TEs is dependent on their propagation through the germline. Thus, the time and place in which they are active is critical to their long-term survival in host genomes. Undifferentiated embryonic cells are one of the “niches” adopted by TEs that facilitate their propagation (Haig 2016). Although the mobility of TEs is thought to be generally deleterious to the host, the accumulation of TEs in the genome represents a source of raw genetic material that may be co-opted during evolution to benefit diverse cellular functions, including functions related to embryogenesis (Lu et al. 2014; Wang et al. 2014; Durruthy-Durruthy et al. 2016; Garcia-Perez et al. 2016; Jachowicz et al. 2017). Zebrafish, a powerful model organism to study embryonic development, is also notable for its very high TE and repetitive DNA content (53%) (Howe et al. 2013) compared with other teleost fish: ∼5% in pufferfish and ∼25% in Mexican tetra (Chalopin et al. 2015; Shao et al. 2019). As yet, however, little is known about the TE ecosystem of the zebrafish genome. Are TE families uniformly distributed across the genome or do they preferentially accumulate in certain regions? What is the demographic profile of zebrafish TEs? Does the diversity of zebrafish TE families result in distinct spatial and temporal patterns of expression during development? Are these expression patterns related to the intrinsic properties of individual TEs or are they driven by their genomic locale? In this work, we aim to answer these questions in order to establish the groundwork for the study of TEs in zebrafish development.

TEs exploit a variety of transcriptional and translational mechanisms to expand in the host genome. Based on their transposition intermediates, TEs are classified as retrotransposons or DNA transposons (Finnegan 1989; Wells and Feschotte 2020). Retrotransposons reverse-transcribe their own RNA and then insert the DNA copy back into the genome. Most retrotransposons carry internal promoters with *cis*-regulatory sequences that recruit the host transcriptional machinery to drive their own expression, much like host genes (Bowen et al. 2003; Romanish et al. 2007; Faulkner et al. 2009; Robbez-Masson and Rowe 2015; Brind'Amour et al. 2018). In contrast, most DNA transposons directly excise themselves and reinsert elsewhere in the host genome, a process mediated by transposase genes encoded by autonomous DNA transposons (Spradling et al. 2011; Fricker and Peters 2014; Hickman and Dyda 2016). Compared with retrotransposons, the mechanisms directing the expression of DNA transposons are generally less characterized. Some do contain promoter sequences, but these tend to be weak and not cell type–specific (Palazzo et al. 2017, 2019). Furthermore, the relative abundance and diversity of DNA transposons and retrotransposons also differ between species. For example, although TEs comprise approximately half of both human and zebrafish genomes, retrotransposons account for ∼95% of all TEs in the human genome but only ∼10% in zebrafish (International Human Genome Sequencing Consortium 2001; Howe et al. 2013). In contrast, ∼40% of the zebrafish genome comprises DNA transposons, whereas in humans, they occupy just ∼3% (Pace and Feschotte 2007). Overall, all the major lineages of eukaryotic transposons, including the rarer types, can be found within the zebrafish genome, which harbors a much greater diversity of TEs than is typically observed in mammalian genomes (Furano et al. 2004; Howe et al. 2013; Chalopin et al. 2015).

Genome-wide studies have revealed that TEs are expressed in a tightly regulated fashion during mammalian embryonic development. In human and mouse early embryos, TE transcripts comprise up to 15% of the transcriptome (Peaston et al. 2004; Svoboda et al. 2004; Göke et al. 2015). Although the expression pattern and regulatory activities of TEs during development likely reflect how they have exploited distinct cellular niches to propagate, these activities may also be integrated in normal developmental programs. For example, the expression of the murine long interspersed nuclear element-1 family (LINE-1) can be detected shortly after fertilization and peaks at the two-cell stage in mouse embryos, while cells are still totipotent (Peaston et al. 2004; Fadloun et al. 2013). This expression not only promotes LINE-1 transposition in mouse early embryos (Richardson et al. 2017) but also, provocatively, may be essential for proper embryonic development (Jachowicz et al. 2017; Percharde et al. 2018). Endogenous retroviruses (ERVs), which are affiliated with LTR retrotransposons, are also transcriptionally active in a highly stage-specific manner in mammalian embryos (Göke et al. 2015; Grow et al. 2015). The expression of MERVL, a murine-specific ERV family, peaks at the two-cell stage of embryogenesis and contributes to the expression of more than 50 chimeric MERVL–host gene transcripts in the mouse embryo (Peaston et al. 2004; Macfarlan et al. 2012). Similarly, HERVH, a primate-specific family, is specifically expressed from the eight-cell to the blastocyst stage and marks cells with higher pluripotent potential (Fort et al. 2014; Wang et al. 2014, 2016; Göke et al. 2015). Thus, understanding TE expression is important for understanding not only the biology of TEs but also that of the host. However, most of what we know about the transcriptional activity of vertebrate TEs during embryogenesis comes from studies conducted in human or mouse, which harbor a very limited diversity of TEs relative to other vertebrates and, indeed, most animals (Wells and Feschotte 2020).

Little has been reported about the expression of TEs in zebrafish, but a few families have been serendipitously identified as markers of specific stages of embryonic development. For example, BHIKHARI, a zebrafish ERV family, is expressed exclusively in the mesendoderm lineage during gastrulation (Vogel and Gerster 1999; Chen and Schier 2001). A distantly related ERV, *crestin* (also known as *BHIKHARI-2*), was discovered as a specific marker of the neural crest (Rubinstein et al. 2000; Luo et al. 2001). Despite these observations, BHIKHARI elements have not been characterized further, and there is a general dearth of information regarding the genomic characteristics and expression of individual TE families in zebrafish. Previous studies examining zebrafish TEs on a genome-wide scale have been limited to broad patterns at the level of TE classes or subclasses (e.g., LTR, LINE, etc.) (Chalopin et al. 2015; Gao et al. 2016; Yang et al. 2020). However, different TE families within the same TE class can behave very differently when it comes to their genomic distribution or expression patterns (Feschotte et al. 2002; Ishiuchi et al. 2015; Rodriguez-Terrones and Torres-Padilla 2018; Stitzer et al. 2021).

To establish a foundation for future work on the activity of TEs in zebrafish embryogenesis, we have performed a detailed characterization of the genomic landscape and embryonic expression of zebrafish TEs. Our study highlights the staggering diversity of TEs in zebrafish, yields insights into the effect of selection on the genomic distribution of different TE types, and describes a wide diversity of transcriptional patterns through early development.

## Results

### The genomic landscape of zebrafish TEs

**Figure 1.**
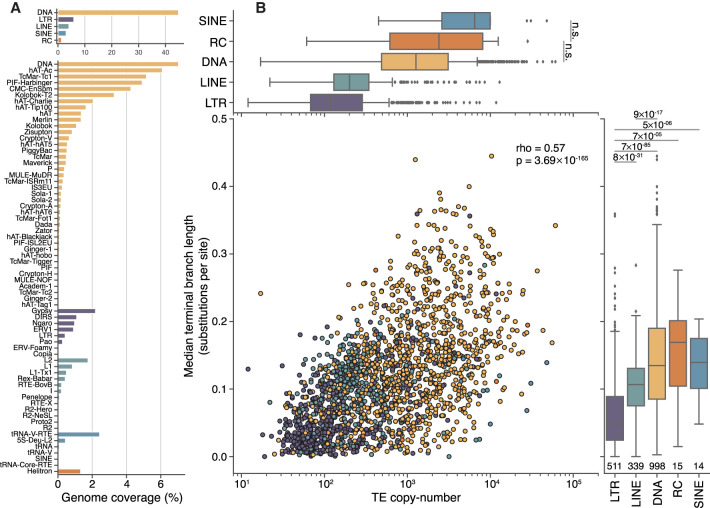
Genome proportions, copy number, and median age differ between TE classes. (*A*) DNA transposons, including rolling-circle elements (Helitrons), take up approximately four times more genomic space than retroelements and contain a greater number of distinct superfamilies. (*B*) Overall, there is a moderate correlation between the copy number of TE families and their median age (Spearman's ρ = 0.57, *P* = 3.69 × 10^−165^). LTR elements, on average, are younger than other classes (lower values on the *y*-axis), and DNA transposons are typically older. Numbers *underneath* the box plots are the number of distinct TE families used in this analysis. Significance was calculated using Wilcoxon rank-sum tests between each TE class, using a Bonferroni-corrected *P*-value threshold of 0.001 for determining significance. For clarity, only the two nonsignificant tests are shown in the *top* panel.

Using RepeatMasker to annotate the *Danio rerio* reference genome (GRCz11), we mapped the location of sequences related to a total of 1931 nonredundant TE families cataloged in Dfam and Repbase (Bao et al. 2015; Storer et al. 2021). These families include representatives of all major classes and subclasses of eukaryotic TEs, including LTRs, non-LTRs (LINEs and SINEs), and tyrosine recombinase retroelements, as well as DDE-type DNA transposons, rolling-circle (RC) elements (i.e., Helitrons), Mavericks/Polintons, and Cryptons (Wicker et al. 2007; Wells and Feschotte 2020). Collectively, interspersed TEs account for 59.5% of the genome, with DNA transposons accounting for 46.2% and retroelements 13.2% (Fig. 1A; Supplemental Data 1). Note that these values are higher than previously reported, likely as a result of improvements in the quality of the zebrafish reference genome since its initial publication (Howe et al. 2013; Howe 2020). Among retroelements, the genome proportion of LTRs, LINEs, and SINEs is 6.0%, 4.1%, and 3.1%, respectively, whereas tyrosine recombinase–mediated retroelements (DIRS and Ngaro superfamilies) account for 2.1%. DNA transposons are dominated by DDE-type transposons, which comprise 43.5% of the genome, whereas the more exotic Helitrons, Cryptons, and Maverick/Polinton elements make up 1.3%, 0.9%, and 0.5%, respectively.

### DNA transposons tend to be older and more abundant than retroelements

We estimated the age of TEs by generating phylogenetic trees for all families with at least 10 copies, using defragmented insertions of at least 100 bp in length (n = 1880) (Supplemental Data 2), and then calculated the median length of terminal branches for each family (measured in nucleotide substitutions per site). This measure correlates well with estimates calculated using divergence from family consensus sequences but avoids biases caused by family substructure (Supplemental Fig. 1; Stitzer et al. 2021). Based on the presence of many families with identical insertions across the genome (i.e., branch length = 0), we can infer that all of the major TE classes in zebrafish—with the possible exception of SINEs, of which there are only 14 annotated families—contain either recently or currently active families (Supplemental Data 3).

Using this measure of age, we observed a moderate positive correlation between the average age of TE families and their copy number (Spearman's ρ = 0.57, *P* ≈ 0) (Fig. 1B). There are very few examples of low copy number elements that are also old; for example, of families with fewer than 50 copies, just three have a median branch length greater than 0.1 substitutions per site. In contrast, there are 45 very young families (fewer than 0.05 substitutions per site on average) with more than 1000 copies, and it is therefore likely that there are many families transpositionally active in zebrafish populations. We also observe significant differences in age and copy number between TE classes: DNA transposon families are typically older and present at a higher copy number than both LINE and LTR retroelement families (Fig. 1). This trend could indicate either (1) a recent increase in the rate of activity of retroelements relative to DNA transposons or (2) differences in the rate at which DNA transposons and retrotransposons are fixed in the population or deleted after insertion (Frahry et al. 2015; Kapusta et al. 2017).

### Differential retention of TE insertions among classes

The rate at which TE insertions are removed by purifying selection is in part determined by the magnitude of their deleterious effects. Because ectopic recombination between TE copies is thought to be a major driver of selection against TEs (Petrov et al. 2003; Boissinot et al. 2006; Blass et al. 2012), the increased turnover of LTRs and LINEs could be driven by selection owing to their greater length relative to DNA transposons, because longer elements provide larger targets for ectopic recombination, all other factors being equal. To investigate this hypothesis, we first confirmed that there are differences in consensus sequence length between the major TE classes represented in the zebrafish genome (Supplemental Fig. 2A). On average, zebrafish LTR elements are approximately 1.3 times longer than LINEs and 4.8 times longer than DNA transposons. We then tested to see if there was a relationship between the consensus sequence length of TE families and their median age and found a moderate, but significant, negative correlation between the two (Spearman's ρ = −0.35) (Supplemental Fig. 2B). These correlations hold when analyzing each class separately, and thus, the relationship between length and age is independent of potentially confounding differences between classes. This result is consistent with a scenario in which longer TE insertions are removed from the zebrafish genome at a faster rate than shorter insertions.

### Genomic distribution of TEs is nonrandom

**Figure 2.**
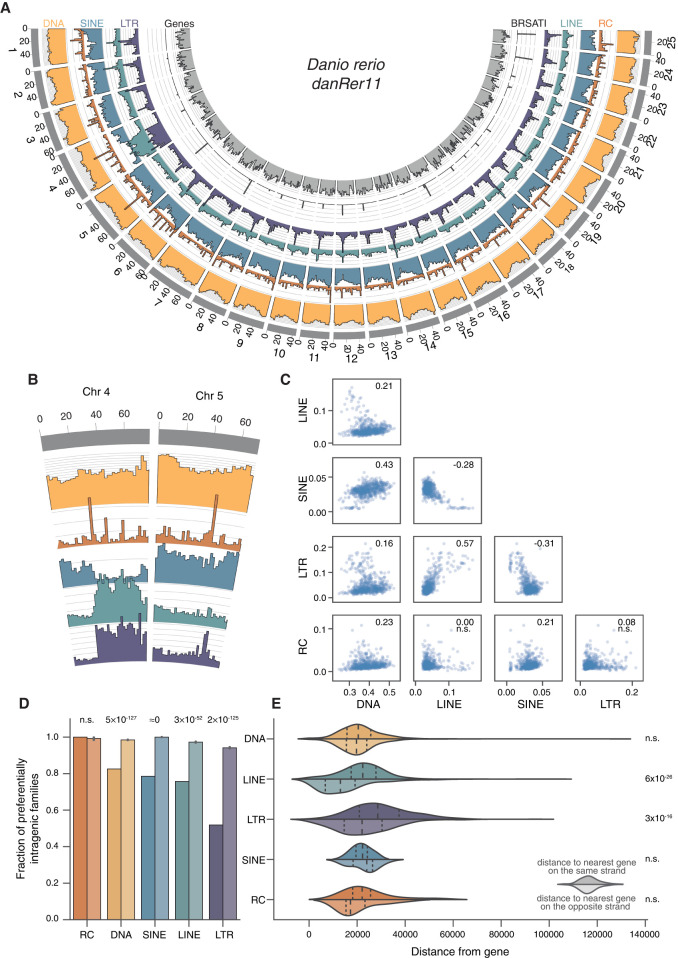
Genomic distribution of elements is nonrandom. (*A*) Genomic coverage of TEs in nonoverlapping 2-Mbp windows across nuclear chromosomes. Each axis line (faint gray) represents 2.5% sequence coverage. (*B*) Detail on Chromosomes 4 and 5. (*C*) Spearman's rank correlations of coverage density between major TE classes. Values for ρ given in *top right* corner of each plot; (n.s.) not significant. (*D*) TE families are defined as “preferentially intragenic” if the median distance between their insertions and the closest gene is zero; that is, most insertions in the family overlap partially or fully with gene bodies. Bars for each TE class represent observed fractions (*left* bars) and fractions based on random shuffling of TE insertion identities across the genome, keeping locations fixed (*right* bars, color desaturated). *P*-values calculated using binomial tests. (*E*) Median, per family, distance of insertions from nearest genes. *Top* halves indicate distance from closest gene on same strand; bottom halves (desaturated), distance from closest gene on opposite strand. *P*-values calculated using Wilcoxon rank-sum tests.

We next looked at the distribution of TEs across chromosomes (Fig. 2A; for details, see Fig. 2B). Visual inspection of TE density plots reveals notable patterns in the distribution of different classes, such as the localized density peaks of RC elements (which may reflect their tendency to form tandem arrays) (Pritham and Feschotte 2007; Thomas et al. 2010), co-enrichment of LTR elements and LINEs, and a negative correlation between LTR/LINE and SINE density. To quantify these observations, we calculated the density (as genome sequence coverage) of different TE classes in nonoverlapping 2-Mb windows along the genome and then calculated the pairwise correlation between groups of interest. This approach reveals significant correlations, both positive and negative, between different TE classes (Fig. 2C). LTR and LINE density is positively correlated (Spearman's ρ = 0.55), whereas SINE density is negatively correlated with both LINE and LTR densities (Spearman's ρ = −0.28 and −0.45, respectively). Similar patterns of opposing LINE/SINE density have been observed in the human, mouse, and rat genomes, although the cause of this phenomenon is not fully understood (International Human Genome Sequencing Consortium 2001; Medstrand et al. 2002; Mouse Genome Sequencing Consortium 2002; Gibbs et al. 2004).

Because LTRs and LINEs accumulate in one particularly dense cluster within each chromosome (Fig. 2A), we reasoned that these could correspond to pericentromeric regions. To corroborate this idea, we compared the density of the satellite repeat BRSATI, a marker of pericentromeric DNA (Phillips and Reed 2000; Howe et al. 2013) to that of LTR and LINE. We found that both LTR and LINE densities were positively correlated with BRSATI density (Spearman's ρ = 0.26, *P* = 5.3 × 10^−12^ and ρ = 0.20, *P* = 7.2 × 10^−8^ for LTRs and LINEs, respectively). Thus, LTR and LINE achieve their highest density in pericentromeric regions. Also of note is the enrichment of retroelements on the long arm of Chromosome 4 (4q), as previously observed (Howe et al. 2013). Because this region is large and thus may be driving some of the observed correlations between the density of TE classes, we repeated the analyses with Chromosome 4 omitted but observed no substantive changes in effect size or significance (Supplemental Fig. 3).

Patterns in chromosomal TE distributions are shaped both by the insertion site preference of the TEs and by natural selection acting after insertion to differentially retain elements inserted in various genomic locations. To disentangle these effects, we regenerated the Circos density plot shown in Figure 2A using only insertions <1% diverged from their family consensus sequence (i.e., young), and those >15% diverged (i.e., old) (Supplemental Fig. 4A,B). Looking at the distribution of young insertions, we see not only that there is still an abundance of LTR and LINE elements in pericentromeric regions and Chromosome 4q but also that the density of young DNA element insertions is also much higher on Chromosome 4q than elsewhere in the genome. In contrast, older insertions of any class are depleted on Chromosome 4q (Supplemental Fig. 4B). These results suggest that the enrichment of TEs on Chromosome 4q may reflect preferential insertion of TEs on this chromosome arm and/or the fact that TEs turn over more rapidly on this arm than elsewhere in the genome.

We next examined the distribution of TE families relative to genes (Fig. 2D). The zebrafish genome is relatively gene dense, with ∼60% of the chromosomal DNA comprising genic regions (∼3% for protein coding sequence), as defined by full-length Ensembl gene annotations on assembled chromosomes. Thus, in the absence of insertion site preference or selection, we would expect the majority of TEs to overlap genic regions. To test whether or not differences exist in the retention of different TE classes across different genic compartments, we categorized each TE family as being either preferentially intragenic if >50% of its copies overlapped with gene bodies or preferentially intergenic otherwise. Then, for each TE class, we calculated the fraction of TE families categorized as preferentially intragenic and compared this to the fraction based on random shuffling of TE identities. With the exception of RC families (n = 16), all families were significantly less likely to be preferentially intragenic than expected, consistent with selection against insertion within genes (Fig. 2D).

To further investigate the distribution of TEs relative to genes, we looked in more detail at intergenic insertions. For each family, we measured the median distance of intergenic insertions to the nearest gene on the same strand, as well as on the opposite strand (Fig. 2E); we find that LTR elements and LINEs are located significantly further away from genes on the same strand than those on the opposite strand (Wilcoxon rank-sum tests: *P* = 3 × 10^−16^ and *P* = 6 × 10^−26^, respectively). Autonomous retroelements often encode strong *cis*-regulatory sequences capable of affecting nearby gene expression, including promoters, splice sites, and polyadenylation signals (Ishiuchi et al. 2015; Clayton et al. 2020; Ng et al. 2020). Thus, there may be stronger selection against zebrafish LTR elements and LINEs when they insert on the same strand as a nearby gene, similar to what has been observed in mammalian genomes (Medstrand et al. 2002).

### Stage-specific regulation of TEs during early development

To investigate TE expression during zebrafish development, we took advantage of a publicly available RNA-seq data set covering 18 stages from one-cell to 5 d post fertilization (White et al. 2017). This high-quality poly(A) pull-down stranded data set, with five biological replicates per time point, constitutes an ideal resource to examine gene and TE expression during early development at a high temporal resolution. To evaluate TE expression, we benefitted from the recent development of computational tools that allowed us to analyze expression of individual TE loci. To do so, we used STAR (Dobin et al. 2013) to map RNA-seq reads to the genome and Telescope (Bendall et al. 2019) to quantify the amount of reads mapping to individual TE copies.

**Figure 3.**
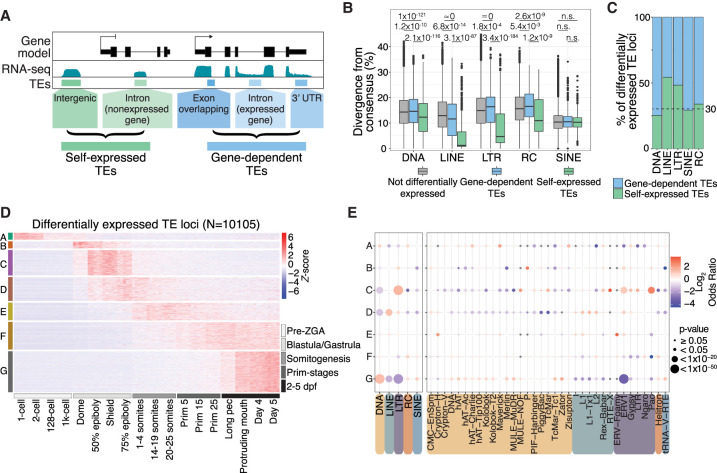
TEs are expressed in stage-specific patterns during zebrafish development. (*A*) Schematic representation of self-expression or gene-dependent expression of TE loci. (*B*) TEs that are both differentially expressed and self-expressed are younger, with lower divergence from consensus, compared with differentially expressed gene-dependent TEs and nondifferentially expressed TEs (to see the divergence from consensus for all TE categories shown in *A*, see Supplemental Fig. 5A). *P*-values were calculated using Wilcoxon rank-sum tests. (*C*) Fraction of differentially expressed gene-dependent or self-expressed TE loci, split by TE class (for split by TE family, see Supplemental Fig. 5C). (*D*) *Z*-score from whole-embryo RNA-seq data (White et al. 2017) shows a subset of differentially self-expressed TE loci displaying stage-specific expression. Clusters are derived using *k*-means clustering. (*E*) TE class-specific (*left*) and superfamily-specific (*right*) enrichment analysis per expression cluster in *D*. Only TE superfamilies with significant enrichment are shown. Gray dots indicate not significant. dpf: days post fertilization.

TEs are abundant throughout the genome and can be incorporated into gene transcripts, for example, through integrations overlapping coding sequences and UTRs (Kelley and Rinn 2012; Wang et al. 2014; Attig et al. 2019) or as a result of intron retention events (Zaghlool et al. 2013). In such cases, it can be challenging to determine if a TE-mapping read originates from a gene promoter or a TE promoter (Lanciano and Cristofari 2020). To address this issue, we categorized the TE annotation based on the TE position with respect to genes (Fig. 3A; Supplemental Data 4). Reads mapping to TEs overlapping annotated exons, UTRs, or introns of expressed genes in the same orientation were considered as transcribed in a gene-dependent manner. These TE-containing transcripts are likely to originate from the host gene's promoter. Conversely, reads mapping to intergenic TEs or TEs in introns of genes that were not detected as expressed in any sample were considered to be driven by their own promoter, or self-expressed (see Methods) (Fig. 3A).

To validate our gene-dependent and self-expressed annotations, we made use of CAGE-seq data from dome and shield developmental stages to detect transcription start sites (TSSs) originating from within TE loci. Comparing gene-dependent loci to those that were both self-expressed and differentially expressed, we found that the latter were strongly enriched for TSSs, being more than 100 times more likely to contain a CAGE-seq peak than gene-dependent loci (*P*-value < 1 × 10^−50^, Chi-square approximation to Fisher's exact test) (Supplemental Fig. 6). We also note that self-expressed TEs (LTR and LINE in particular) were found to be generally younger than gene-dependent TEs (Fig. 3B; Supplemental Fig. 5A), consistent with degradation of promoter functionality over time (Chuong et al. 2017).

We observed a high number of alternative transcription termination sites that were not annotated in the reference transcriptome (GRCz11.98) (Supplemental Fig. 7A). To prevent a TE embedded within these extended 3′ UTRs from being categorized as self-expressed, we used a transcript assembly strategy to capture all the extended 3′ UTRs (see Methods). TEs overlapping these extended 3′ UTRs were considered gene-dependent and were not included for further analysis. We note that our extended 3′ UTRs strongly coincide with the revised gene annotations recently reported by Lawson et al. (2020) (Supplemental Fig. 7B,C). Overall, we determined that self-expressed TE-derived reads account for ∼0.6% and 2.5% of the reads in pre-ZGA and post-ZGA stages, respectively (Supplemental Fig. 5D; Supplemental Data 5). Together, these filtering strategies ensure that the subsequent TE differential expression analysis highlights changes in the expression derived from the direct regulation of TEs rather than differences in the expression of their surrounding genes.

We then conducted a time course differential expression analysis to detect TEs that are transcriptionally regulated during development. To do so, we performed pairwise comparisons across all developmental stages and identified differentially expressed TEs as those with an FDR-adjusted *P*-value < 0.01 in any comparison. Notably, from all differentially expressed TE loci, 32% were self-expressed (Fig. 3C), whereas the rest were gene-dependent, highlighting the importance of differentiating these two categories. Clustering of expression profiles for self-expressed TEs revealed distinct temporal clusters, suggesting that TE expression is tightly regulated during zebrafish development (Fig. 3D). A subset of 466 self-expressed TE loci (4.6%) is detectable at the zygote and two-cell stages but is silent throughout the rest of development (Fig. 3D, cluster A). Because the zebrafish embryo is transcriptionally inactive at this stage (Heyn et al. 2014), these TE transcripts are likely to be maternally deposited during oocyte maturation and subsequently degraded after zygotic genome activation (ZGA). Post ZGA, different TEs show stage-specific expression patterns spanning from the dome stage until 5 d post fertilization (Fig. 3D). Notably, clusters B (469, 4.6%) and C (1720, 17%) define two subsets of TE loci sharply activated post-ZGA and up-regulated during the blastula/gastrula stages. Clusters D (1608, 15.9%) and E (1195, 11.8%) contain TE loci that are up-regulated later in development, during somitogenesis, whereas clusters F (1870, 18.5%) and G (2777, 27.5%) mark a group of TE loci that peak in expression during later stages. Together, these data suggest that many TEs are expressed in a tightly regulated manner during zebrafish embryonic development.

Next, we performed an enrichment analysis to detect over- or underrepresented TE classes and superfamilies within each TE expression cluster (Fig. 3E). DNA transposons were generally enriched in clusters with late (larval) expression and depleted in clusters corresponding to the blastula and gastrula stages. In contrast to DNA transposons, retroelements, and LTR elements in particular, were generally enriched in clusters marking earlier developmental stages (Fig. 3E). Specifically, the LTR superfamilies ERV1, Gypsy, and Pao were enriched within cluster C, which marks early post-ZGA expression at the blastula/gastrula stages. Most LINE superfamilies (I, L1, Tx1, and L2) were enriched within cluster D, which corresponds to the late stage of gastrulation and early somitogenesis (Fig. 3E).

To further test the biological interpretation of these results, we reanalyzed ATAC-seq data (Marlétaz et al. 2018) to assess the chromatin accessibility of members of the cluster C-enriched ERV1 superfamily (Fig. 3E). Comparing different developmental stages, we find that opening of chromatin over ERV1 loci coincides with the timing of expression as measured from RNA-seq (Supplemental Fig. 8). Finally, we used the retrotransposon ERV1-N6 as a case study to link host transcription factors to transcription of self-expressed TEs. Focusing on the transcription factor *nanog-like* (Xu et al. 2012), ChIP-seq data show that 61 out of 188 self-expressed ERV1-N6 loci are clearly bound by Nanog-like (Supplemental Fig. 9A,B). Upon close inspection of the LTRs, we find Nanog-like binding motifs directly upstream of the TSS and TATA box predicted for these elements (Supplemental Fig. 9C). Thus, ATAC-seq and ChIP-seq data support the notion that self-expressed TEs contain functional promoter sequences. Together, these results suggest that different TE classes and superfamilies have distinct expression profiles during zebrafish development, including a pronounced activation of LTR retroelements shortly after ZGA, when early cell fate decisions are made.

### Single-cell RNA-seq resolves somatic TE expression during early embryogenesis

Our analysis of whole-embryo RNA-seq data suggests that many zebrafish TE families display stage-specific expression patterns during embryonic development. To investigate cell type–specific and lineage-specific TE expression during early development, we turned to a publicly available single-cell RNA sequencing (scRNA-seq) data set (Farrell et al. 2018). This data set spans 12 developmental stages, ranging from 3.33 h post fertilization (hpf; the so-called high stage) to 12 hpf (six-somite stage), allowing us to track TE expression along specific developmental trajectories. We realigned scRNA-seq reads to the most recent zebrafish genome assembly (GRCz11) and annotated reads to both genes and self-expressed TE loci defined from our bulk RNA-seq analysis. After data processing, we excluded potential cell doublets and cells with low complexity transcriptomes and high proportions of mitochondria RNAs (Farrell et al. 2018). We were left with data spanning 45,127 cells across all stages. Approximately 2.9% of reads mapped to self-expressed TE loci, which is comparable with what we observed with the whole-embryo RNA-seq data. Because of the shallow sequencing depth of scRNA-seq and the repetitiveness of TEs, it is difficult to confidently assess expression at individual TE loci (He et al. 2021; Shao and Wang 2021). Thus, we analyzed the expression profile of TEs at the family level by counting all reads mapping to loci from the same TE family.

**Figure 4.**
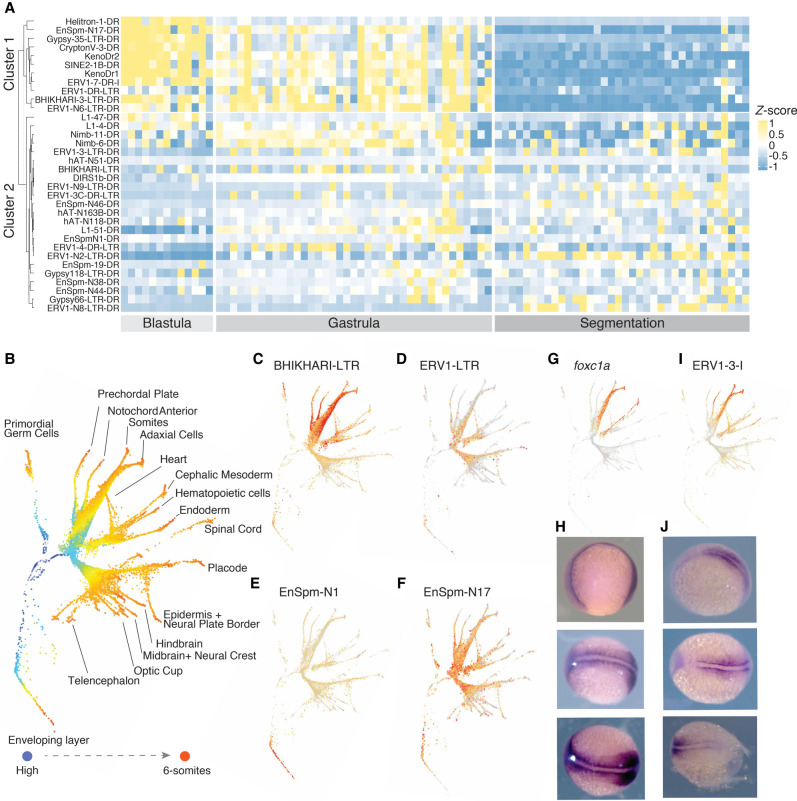
TE families with cell lineage–specific expression across development stages. (*A*) Heatmap of differentially expressed TE families between cell clusters across developmental stages. Hierarchical clustering shows two groups of TE families with distinct expression patterns: one group with early expression in the blastula and gastrula stages and one group with later expression in the gastrula and segmentation stages. TE classes are equally represented in both groups. (*B*) Pseudotime tree across 12 development stages based on both gene and TE expression. (*C*–*F*) TE families with expression patterns in different cell lineages: (*C*) BHIKHARI-LTR, (*D*) ERV1-LTR, (*E*) EnSpm-N1, (*F*) EnSpm-N17. (*G*,*H*) The expression pattern of *foxc1a* in a pseudotime tree (*G*) and in 11 hpf embryos by in situ hybridization (*H*). (*I*,*J*) The expression pattern of ERV1-3-I in the pseudotime tree (*I*) and in 11 hpf embryos by in situ hybridization (*J*). hpf: hours post fertilization.

To identify TE families expressed in specific cell types, we grouped all cells across the 12 stages into 63 cell-type clusters based on both gene and TE family expression (see Methods) (Supplemental Data 6). To validate these clusters, we verified that they had captured known marker genes for distinct cell lineages, such as primordial germ cells (PGCs), the enveloping layer, and notochord cells (Supplemental Fig. 10A; Farrell et al. 2018), and correctly separated cells based on their developmental stages (Supplemental Fig. 10B). To identify differentially expressed TE families between cell clusters, we compared TE expression levels within each cluster to their expression levels in the rest of the cells. To avoid possible technical noise from scRNA-seq (Farrell et al. 2018; Shao and Wang 2021), we focused on TE families that are expressed in >20% of cells in at least one cell cluster, using only reads mapping to the self-expressed loci identified from bulk RNA-seq. Among those, 34 TE families were significantly up-regulated in at least one cell cluster compared with all other cells (Fig. 4A). We repeated this analysis using only uniquely mapping reads and found only four families with lineage-specific expression patterns. This is to be expected, as TE families in zebrafish tend to be young (Fig. 1B) and thus less likely to be detected with uniquely mapping reads.

Using hierarchical clustering analysis, these families can be divided into two broad categories: (1) a group of 11 TE families that are highly expressed in the blastula (largely undifferentiated cells) and gastrula stage but not later in development and (2) a group of 23 TE families that are expressed much later in development, when most cells have already differentiated into distinct cell lineages. In agreement with our bulk RNA-seq results for retrotransposons, the first group (early expression) includes members of the Gypsy, ERV, and L1 superfamilies and a few members of the EnSpm (CMC), Helitron, and Crypton superfamilies of DNA transposons. The second group (late expression) includes representatives from most the retrotransposon superfamilies identified in the first group but only EnSpm and hAT DNA transposons. We found no significant differences in age or TE classes between those two broad groups of elements (Mann–Whitney *U* test, *P* = 0.18). Overall, TE families belonging to the ERV1 superfamily were strongly enriched across both groups (11 out of 34 TE families) compared with their representation in the genome (54 out of 1931 TE families, Fisher's exact test, *P* < 0.001).

To visualize TE families with late expression patterns along developmental trajectories, we conducted a pseudotime tree analysis (Fig. 4B; Farrell et al. 2018). This revealed that several TE families are highly expressed in specific somatic cell lineages (Fig. 4D–F). One such TE is BHIKHARI, an ERV1 family expressed exclusively in the mesendoderm and in PGCs (Fig. 4C). These results corroborate earlier reports that BHIKHARI transcripts specifically mark the developing mesendoderm of zebrafish (Vogel and Gerster 1999). Furthermore, we note that BHIKHARI expression is driven by the majority of BHIKHARI copies (97 out of 98 self-expressed BHIKHARI loci) dispersed throughout the genome and not by a single or a few isolated copies (Supplemental Fig. 11A).

Another example is ERV1-3, which is highly expressed in the axial and paraxial mesoderm after the 50% epiboly stage (Fig. 4I). Again, we found that ERV1-3 expression was driven by multiple insertions in the genome (71% of reads were from 10 out of 45 self-expressed loci) (Supplemental Fig. 11B), suggesting that this expression pattern is driven at least in part by ERV1-3's own promoter activity rather than the local genomic environment. To experimentally validate these observations, we conducted in situ RNA hybridization using a probe designed against the *pol* gene of ERV1-3 on embryos at the three-somite stage (11 hpf). As a comparison, we also performed in situ hybridization with a probe for *foxc1a*, a transcription factor known to mark the paraxial mesoderm (Fig. 4G; Topczewska et al. 2001; Wilm et al. 2004). The results show that the ERV1-3 and *foxc1a* RNA transcripts have very similar expression patterns in zebrafish embryos, and both specifically mark the paraxial mesoderm (Fig. 4H–J). Our in situ validation of ERV1-3 also highlights the importance of including multimapping reads when analyzing TE expression: 95% of the ERV1-3-derived reads in the Farrell et al. (2018) data set are multimappers, and when we restricted our analysis to uniquely mapping reads, differential ERV1-3 expression was no longer detectable.

Although transcripts of DNA transposons are mostly driven by nearby genes (Fig. 3C), several families are self-expressed and differentially expressed between cell clusters. Among those, members of the EnSpm superfamily were enriched in our analysis (Fisher's exact test, *P* = 0.0352). Most of the EnSpm families with lineage-specific expression are nonautonomous elements with no detectable coding sequences. Yet, analysis of individual loci indicates that the expression of each EnSpm family was driven by multiple loci throughout the genome. Together, these data suggest that diverse TE families encompassing both retrotransposons and DNA transposons display a specific pattern of spatiotemporal expression in developing zebrafish embryos.

## Discussion

In this work, we have performed a comprehensive analysis of the zebrafish TE ecosystem and their embryonic niche using a wealth of transcriptomic data spanning developmental stages from pre-ZGA to 5 d post fertilization. The zebrafish genome contains nearly 2000 TE families from all major classes and superfamilies, ∼65% of which are expressed during development. From analyses of both bulk and single-cell expression data, our results suggest that zebrafish TEs span a wide diversity of expression patterns, from highly stage-specific and cell type–specific expression to broad expression throughout development. These patterns vary both between TE classes and within superfamilies and are in part reflected in the broad differences in their genomic distribution.

Measuring the expression of TEs remains a challenge in genomic analyses owing to their repetitive nature, intricate transcriptional relationship with host gene expression, and the general complexity of the transcriptome (Lanciano and Cristofari 2020). Short reads mapping to TE sequences cannot easily distinguish whether they derive from a TE promoter or are part of a gene or read-through transcript of sorts, including noncoding RNAs, which are ubiquitous in vertebrate genomes (Kung et al. 2013) and often contain TEs (Kapusta et al. 2013). Recent studies have attempted to address these technical difficulties by analyzing exon-overlapping, intronic, and intergenic reads separately, both with bulk and scRNA-seq data (Kong et al. 2019; He et al. 2021; Shao and Wang 2021). In this work, we have combined Telescope, a recently developed tool to detect TE expression at single-locus resolution from bulk RNA-seq data (Bendall et al. 2019), with genome-based classification to differentiate between TE expression most likely derived from gene promoters or from TE promoters (see Methods) (Fig. 3A). This approach suggests that around two-thirds of TE-mapping reads in the zebrafish transcriptome are most likely associated with host gene expression and read-through transcription. Thus, the majority of TE sequences in zebrafish are not expressed from their own promoters but are expressed as part of chimeric read-through transcripts, both coding and noncoding.

TE fragments embedded in gene transcript isoforms may have diverse functional consequences. For example, they have been shown to be the source of new protein coding exons, RNA-binding motifs, and microRNA target sites (Lev-Maor et al. 2003; Zarnack et al. 2013; Petri et al. 2019; Cosby et al. 2021). Our analyses reveal that DNA transposon transcription is more often gene-dependent than retroelement transcription. One feature of DNA transposons that may facilitate their hijacking of host promoters is the presence of splice sites within their sequence, which has been implicated in the formation of chimeric transcripts that occasionally encode transposase–host fusion proteins co-opted for cellular function (Cordaux et al. 2006; Newman et al. 2008; Cosby et al. 2021). An interesting case is the Maverick/Polinton class of DNA transposons, which is strongly enriched at zygotic and pre-ZGA stages. Maverick/Polinton elements have been associated with the DNA 6mA modification during early embryonic zebrafish development, hinting at the unusual regulation of this family of TEs at this stage (Liu et al. 2016). We wish to emphasize that our definition of self-expressed TEs is conservative and may underestimate the activity of TE-derived promoters. For example, we noticed that among differentially expressed gene-dependent TE loci, LTRs were the TE class with the highest fraction of overlap with 5′ UTR and coding exons (Supplemental Fig. 4B). These may represent chimeric LTR–host gene transcripts driven by LTR rather than host gene promoters (Thompson et al. 2016). Currently, identifying such chimeric gene–TE transcripts is technically challenging, but long-read transcriptome sequencing will ease many of these difficulties and is therefore a promising avenue for future studies.

Using bulk and single-cell RNA-seq to untangle temporal and lineage-specific patterns of TE expression, we observe broadly distinct patterns between the major TE classes. As documented in mammalian species (Göke et al. 2015; Grow et al. 2015; Franke et al. 2017), we observe that LINE and LTR retroelement transcripts are particularly abundant at or shortly after ZGA in zebrafish. In contrast, we find that DNA transposon transcripts tend to be enriched before ZGA (i.e., maternally deposited) or expressed later in development. We also note that retroelement insertions are significantly more likely to drive their own expression than are DNA elements, which is consistent with the fact that retroelements typically encode strong promoters, whereas DNA transposons are thought to have generally weaker or less specific promoters (Palazzo et al. 2017, 2019). Differences in intrinsic transcription capability affect not only TE expression but also genomic distribution, and the more robust expression of retroelements we observed may also explain why they are less common nearby or within genes than DNA transposons, because their promoters and other *cis*-regulatory elements have greater potential to interfere with gene expression. Similarly, we found that SINEs, which are usually transcribed by RNA pol III and therefore less likely to interfere with pol II–mediated regulation, are also more closely associated with genes than other subclasses of retroelements, a trend also observed in mammalian genomes (International Human Genome Sequencing Consortium 2001; Gibbs et al. 2004).

Differences in genomic distribution may also be partly driven by the length of elements, which is thought to be correlated with the frequency at which they contribute to ectopic recombination (Petrov et al. 2011). Indeed, LINEs and LTR retroelements are generally longer than DNA transposons. Recombination rates across the genome are another important factor influencing TE distribution and likely explain the tendency for LTRs and LINEs to be enriched in pericentromeric regions, in which recombination is suppressed (Kent et al. 2017). This is particularly relevant to Chromosome 4q, which is highly repeat rich, likely owing to a combination of insertion preference of TEs and reduced recombination (Bradley et al. 2011). Thus, the potential to interfere with gene expression and the interplay with recombination act in concert to shape the differential accumulation of zebrafish TE classes relative to genes.

With respect to expression, certain superfamilies stand out in both the bulk and scRNA-seq analyses: most notably the ERV1 superfamily. ERV elements tend to be highly expressed immediately after ZGA, often in a cell type–specific fashion and apparently using their own promoters, before being silenced later in development. This pattern suggests that ERV expression is governed by tightly regulated *cis*-regulatory sequences responsive to both transcriptional activators as well as repressors. This is reminiscent of mammalian ERVs, which are activated by stage-specific TFs and repressed by sequence-specific KRAB-zinc finger proteins (Bruno et al. 2019; Hermant and Torres-Padilla 2021). Given the lack of the KRAB domain in zebrafish, a clear research avenue for the future will be to identify the transcriptional regulators silencing zebrafish ERVs. Compared with other TE types, ERVs appear to be more intimately tied up in the host embryonic development process, and this raises the possibility that they are able to influence embryogenesis to an extent that we have not yet fully appreciated.

Another intriguing finding of our study is the identification of a small subset of TE families (e.g., BHIKHARI and ERV1-3 from the aforementioned ERV1 superfamily) with a high level of RNA expression in somatic progenitor cell lineages. Could such somatic expression facilitate transposition in the germline? One possibility is that somatic expression provides an indirect route for TEs to enter the germline; this has been observed during oogenesis in *Drosophila melanogaster*, where TEs expressed in support cells surrounding the oocytes either infect or are trafficked into mature oocytes (Chalvet et al. 1999; Wang et al. 2018). Alternatively, it may be that somatic expression is only mildly deleterious to the zebrafish host and, therefore, occasionally arises with little functional consequence for either the host or the TEs. This may be the case if the resulting protein products are nontoxic and if somatic transposition events remain rare. Finally, it is possible that spatiotemporal patterns of TE expression may occasionally support organismal development. Although this is a provocative idea, there are now several examples of TEs with important roles in embryonic development: L1 and MERVL in mice (Macfarlan et al. 2012; Jachowicz et al. 2017; Percharde et al. 2018), HERVK and HERVH in humans (Lu et al. 2014; Grow et al. 2015), and ERNI in chickens (Blanc et al. 2014); the last of these is noteworthy as it functions in a strictly somatic niche. Functional experiments will be necessary to determine whether zebrafish TEs expressed in somatic lineages reflect selfish, neutral, or mutualistic behaviors, and we anticipate that this will be a fruitful topic of study in coming years.

The activity of retroelements during early embryonic development has been noted in many vertebrate species, particularly in mammals. Many of the patterns observed in these studies are recapitulated in zebrafish, indicating that features such as robust expression following ZGA, lineage-specific expression, and accumulation in gene-poor regions are features shared by diverse retroelement superfamilies across a broad range of vertebrates. In contrast, much less is known about the behavior of DNA transposons during development, largely owing to the paucity of active DNA transposon families in mammals, with the notable exception of vespertilionid bats (Platt et al. 2016). Unlike LINEs and LTR elements, DNA transposon-derived transcripts are enriched both very early in development (before ZGA) and in the latest stages of development (4–5 dpf). Mechanisms to prevent activation and mobilization of TEs in germ cells, such as the Piwi-interacting RNA (piRNA) pathway, have been described in zebrafish (Houwing et al. 2007, 2008; Kaaij et al. 2013). piRNAs and Piwi proteins are maternally deposited and localized in the germ plasm (Houwing et al. 2007). Following the first cell divisions, cells that inherit the germ plasm will develop into PGCs (Raz 2003). Zebrafish piRNAs are enriched in LTR targets and contain fewer DNA transposon targets, indicating a greater degree of protection against younger LTR elements compared with DNA transposons (Houwing et al. 2007; Kaaij et al. 2013). Thus, the depletion of LTR transcription in pre-ZGA stages, which mainly contain maternally deposited transcripts, may be owing to efficient repression by the piRNA pathway. For example, piRNAs targeting Harbinger DNA transposons are abundant in zebrafish ovaries, possibly explaining the depletion of this superfamily at early stages (Fig. 3D; Houwing et al. 2007).

With its unusually rich TE content, zebrafish, more than most, exemplifies the idea of the genome as an ecosystem. Much like the species they parasitize, TEs possess traits that are shared across taxonomic groups but also traits that are unique to each family. For almost all TEs, however, embryonic development is a critical period for their long-term success, and increasingly, it is clear that many TEs are not idle passengers in the process. Zebrafish are a powerful model for the study of vertebrate embryogenesis and, yet, are only beginning to attract interest as a system for studying genome evolution and the role of TEs during the process. There are many open questions that we have not touched upon in this study, including, but not limited to the following: the effect of TEs on gene regulation, differences in TE activity between sexes, activity in the germline, and inter-species and inter-individual variation in the TE ecosystem. It is our hope that this work provides a useful foundation for investigating these questions and for developing zebrafish as a model for further study on the fascinating interplay between TEs and their hosts.

## Methods

### Transposable element annotation

TEs were mapped to the zebrafish genome (May 2017; GRCz11/danRer11, accessed from the UCSC Genome Browser) using RepeatMasker version 4.08 (Smit et al. 2013–2015; http://www.repeatmasker.org). For mapping, we used the rmblastn engine (version 2.2.27+) and the Dfam_Consensus-20181026 and Repbase-20181026 libraries. The following parameters were set: -a, -s, -nolow, -gccalc, -gff, -cutoff 200, -no_is. The RepeatMasker output files were processed using ParseRM (Kapusta et al. 2017; https://github.com/4ureliek/Parsing-RepeatMasker-Outputs); this was used to generate measurements of Kimura CpG-corrected percentage-divergence from consensus sequence. TE copy number estimates were acquired from the output of the Perl script onecodetofindthemall.pl (Bailly-Bechet et al. 2014), which reconstructs fragmented repeats and full-length LTR elements.

### Dating TE insertions

To build phylogenetic trees for each TE family, defragmented sequences were extracted from the genome and aligned using MAFFT v7.419 (Katoh and Standley 2013), with the ‐‐auto flag set to true. A minimum sequence length of 100 was specified for inclusion in the alignments. Multiple sequence alignments were trimmed using trimAl v1.4.1 (Capella-Gutierrez et al. 2009), with the -gt parameter set to 0.01. TEs with fewer than 10 suitable sequences were ignored, and alignments of TEs with a high copy number were restricted to a random selection of 1250 sequences, in order to enable computation in a reasonable time frame. FastTree v2.1.10 (Price et al. 2010) was used to construct approximate maximum-likelihood phylogenetic trees, using a generalized time-reversible model. Branch lengths were rescaled to optimize Gamma20 likelihoods. For a given TE insertion, the age was specified as the branch length from the leaf to the most recent ancestor (terminal branch length); for a family of TEs, the average age was calculated as the median of the terminal branch lengths. RepeatMasker-derived divergence from consensus sequence was used as an alternative measure of age in Supplemental Figure 3.

### Analysis of genomic distribution

To visualize the genomic distribution of different TE classes, we split the genome into 2-Mbp windows and calculated TE coverage as the percentage of TE-derived base pairs in each window. The results were plotted with Circos (Fig. 2A; Krzywinski et al. 2009). The relationship between the distributions of different TE classes was calculated with Spearman's rank correlation on the windows, sorted by percentage coverage.

To investigate the distribution of TEs relative to genes, the distance between each insertion and the nearest gene was measured using BEDTools (Quinlan and Hall 2010). For a given TE family, if the median distance between insertions and genes was equal to zero, we described that family as “preferentially intragenic.” We then compared the observed fraction of preferentially intragenic families to that expected based on random shuffling of TE labels throughout the genome, thus keeping the overall TE distribution the same but removing differences between families. The significance of the difference between observed and expected intragenic fractions for each TE class was assessed using binomial tests. Last, for each family, we compared the median distance of insertions to genes on the same strand and different strands. We then compared the distribution of these estimates for each TE class, comparing distance on same strand and different strand using Wilcoxon rank-sum tests.

### TE loci classification

ChIPseeker (Yu et al. 2015) was used to annotate the TEs' position with respect to protein-coding genes (GRCz11 annotations, release 98) with the following genomic priority: genomicAnnotationPriority = c(“5UTR,” “3UTR,” “exon,” “promoter,” “intron,” “downstream,” “intergenic”). If a TE was annotated as exonic or in a 5′ or 3′ UTR region, it was considered “exon overlapping.” For intronic TEs, if the residing gene had more than 10 normalized counts in at least one sample, the TE was considered an “intron expressed gene.” On the other hand, if an intronic TE was within a gene that did not have at least 10 normalized counts at any sample, then it was considered an “intron nonexpressed gene.” TEs overlapping extended 3′ UTR regions (see details below) were considered an “extended 3′ UTR.” The rest of TEs were considered “intergenic.” “Exon overlapping,” “intron expressed gene,” and “extended 3′ UTR” were considered gene-dependent TEs. On the other hand, “intron nonexpressed gene” and “intergenic” TEs were considered self-expressed. TE fragments reconstructed as part of the same TE by onecodetofindthemall.pl were given the same TE classification in the following hierarchy: exon > extended 3′ UTR > intron expressed gene > intron nonexpressed gene > intergenic.

### Bulk RNA-seq mapping

RNA-seq data (White et al. 2017) were downloaded from European Nucleotide Archive (ENA; https://www.ebi.ac.uk/ena/browser/home) under accession number ERP014517. Paired-end reads were trimmed using BBduk (http://jgi.doe.gov/data-and-tools/bbtools) with the following parameters: ktrim = r, k = 23, mink = 11, hdist = 1, tbo, tpe. Trimmed reads were mapped to the GRCz11 zebrafish genome with appended ERCC spike-in sequences using STAR (version 2.5.2b) (Dobin et al. 2013) with the following parameters: ‐‐chimSegmentMin 10 ‐‐winAnchorMultimapNmax 200 ‐‐outFilterMultimapNmax 100. STAR genome index was generated giving GRCz11.98 Ensemble annotations with the parameter –sjdbGTFfile. Alignment files were sorted and indexed using sambamba (version 0.6.7) (Tarasov et al. 2015). TEtranscripts (Jin et al. 2015) was run to obtain gene counts with the following parameters: ‐‐stranded reverse ‐‐mode multi. Telescope (Bendall et al. 2019) was used to obtain TE counts at TE-locus resolution. Because Telescope does not consider stranded RNA-seq data, alignment files were split between forward and reverse mapping strand using an ad hoc script with SAMtools (version 1.10) (Li et al. 2009) to subset based on SAM flags. Forward alignment files were counted to forward orientation TEs, and reverse alignment files were counted to reverse orientation TEs using telescope assign (version 1.0.3) with the following parameters: ‐‐theta_prior 200000 ‐‐max_iter 200 ‐‐updated_sam. Counts from TE fragments reconstructed by onecodetofindthemall.pl were merged.

### Bulk RNA-seq differential expression analysis

DESeq2 (version 1.28.1) (Love et al. 2014) was used to perform the differential expression analysis of genes and TE loci. ERCC spike-in mix was used to calculate a normalization factor using RUVSeq (version 1.24.0) (Risso et al. 2014) to remove unwanted variation using the TEtranscripts gene counts. DESeq was run for gene and TE counts together with the following experimental design: spike-in normalization factor + developmental stage. Because gene expression accounts for a bigger fraction of the transcriptome, running DESeq on TE counts together with gene counts ensures a better dispersion estimation that will impact DESeq normalization. To remove nonexpressed genes and TEs, only genes and TEs with more than five reads in at least two samples were considered. To obtain TEs that were differentially expressed during development, pairwise comparisons between any developmental stage were performed. Multiple test correction for all the pairwise comparisons was performed by stacking all the result tables from each comparison in a single table and using p.adjust function with parameter method = “BH” in R (version 4.0.1) (R Core Team 2021) to calculate the adjusted *P*-value. To remove very lowly expressed signal, TEs with fewer than 10 reads in any stage were removed. TEs with a *P*-adjusted value lower than 0.01 in any pairwise comparison were considered differentially expressed. Finally, gene-dependent TE loci were discarded.

### TE loci clustering and enrichment analysis

The differentially expressed TE loci normalized count matrix was standardized using *Z*-score transformation. Then, the matrix was clustered using *k*-means clustering R function kmeans with the following parameters: iter.max = 500, nstart = 50 and algorithm = “Lloyd.” After visual inspection, it was decided to limit the number of clusters to seven because it represented most of the variance without over clustering. Heatmap representation of the matrix was produced using pheatmap function from the pheatmap R package (version 1.0.12). Enrichment of TE classes and families within TE loci clusters was performed using the fisher.test R function for contingency tables build by counting TEs from class X or class not X in cluster Y or not Y. Statistical *P*-values were corrected for multiple testing.

### ATAC-seq data processing

Processed ATAC-seq BAM files mapped to GRCz11 genome for the Marlétaz et al. (2018) study were downloaded from the DANIO-CODE (Hörtenhuber et al. 2020; Baranasic et al. 2021) website (series DCD000433SR). BAM files were sorted by query name (-n) using SAMtools (version 1.10) (Li et al. 2009) and processed using Genrich (https://github.com/jsh58/Genrich, version 0.5_dev) with the following parameters: -t BAM -o OUT_Peak -f OUT_pq -k OUT_bdg -e chrM -j -y. bigWig tracks and heatmaps were produced using the deepTools suite (version 3.5.0) (Ramírez et al. 2016). Nucleosome-free regions tracks were generated using bamCoverage with the NucleosomeFree.bam output file from Genrich and the following parameters: -bs 1 ‐‐extendReads ‐‐skipNonCoveredRegions ‐‐scaleFactor. Scale factor was calculated using multiBamSummary with the bin size of 10 bp and ‐‐extendReads. A heatmap was processed with computeMatrix and plotHeatmap using the following parameters: scale-regions -b 100 -m 1000 -a 1000 -b 1000 ‐‐missingDataAsZero. Mappability scores calculated with GEM library tool (Derrien et al. 2012) were included on the heatmap for clarity.

### ChIP-seq data processing

We explored a publicly available ChIP-seq data set (NCBI Gene Expression Omnibus [GEO; https://www.ncbi.nlm.nih.gov/geo/] under accession number GSE34683) (Xu et al. 2012) focusing on the transcription factor Nanog-like because the binding motif is known in zebrafish. We first mapped reads to the genome using STAR (Dobin et al. 2013, v2.7.5a), excluding multimapping reads (‐‐outFilterMultimapNmax 1 ‐‐alignIntronMax 1 ‐‐alignEndsType EndToEnd) and then created a heatmap using deepTools as for the ATAC-seq data (version 3.5.0) (Ramírez et al. 2016). We assessed the enrichment of TEs loci for Nanog-like binding with permutation tests using the TEanalysis suite (Kapusta et al. 2013; https://github.com/4ureliek/TEanalysis). ERV1-6 loci were aligned using MAFFT v7.419 (Katoh and Standley 2013) and manually inspected to identify Nanog-like binding motifs.

### CAGE-seq data processing

We used publicly available CAGE data sets from dome and shield stages to validate our TE categorization (NCBI BioProject database [https://www.ncbi.nlm.nih.gov/bioproject/] under accession number PRJNA602610) (Pillay et al. 2021). We mapped the reads back to the genome using STAR (version 2.7.5a) (Dobin et al. 2013), as for the ChIP-seq data. Duplicates were removed using Picard tools (v2.19.2, https://github.com/broadinstitute/picard) and peaks called using MACS2 (v2.2.7.1; -p 0.01, ‐‐nomodel, ‐‐keep-dup all, -g 1.5e9) (Zhang et al. 2008). We then intersected CAGE peaks to either gene-dependent or self-expressed loci using BEDTools (Quinlan and Hall 2010), counting only TSS peaks fully contained within TE loci (BEDTools intersect -f 1).

### Detection of extended 3′ UTR regions

StringTie (version 1.3.6) (Pertea et al. 2015) was used to find the extended 3′ UTR regions not present in GRCz11.98 Ensembl annotations. Alignment files from biological replicates were combined to increase sequencing depth. StringTie was run without reference annotations and with the following parameters: ‐‐rf -t -c 1.5 -f 0.05. Using an ad hoc R script, for each isoform of known genes, the last exon was subtracted, and using GRCz11.98 Ensembl annotations, the 3′ extended region was calculated. Extended 3′ regions were calculated separately for each developmental stage and collapsed into a single annotation. Extraction of extended 3′ UTR regions with respect to GRCz11.98 Ensembl annotations was performed similarly for data from Lawson et al. (2020) in order to compare them with this study's extended 3′ UTR regions.

### Mapping and annotation of the single-cell RNA-seq data

We downloaded the single-cell RNA-seq data from Farrell et al. (2018) and remapped the reads to GRCz11/danRer11 using Bowtie 2 (Langmead and Salzberg 2012). We used the same parameters for Bowtie 2 as described by Farrell et al. (2018). After mapping to the reference, we annotated reads to both genes and TEs using Drop-seq tools as in the Drop-seq Alignment Cookbook v2.0.0 (Macosko et al. 2015). The reference file of genes for GRCz11 was downloaded from Ensembl (GCA_000002035.4). TE reference file was created from RepeatMasker v4.08 as described, and we only annotate TE transcripts to self-expressed TE loci identified from our bulk RNA-seq analysis. After the annotation, we combined all the reads from the same TE family and only counted the expression level at TE family level. We then created a matrix of digital gene expression (DGE) for both genes and TE families using the DigitalExpression function in Drop-seq tools (Macosko et al. 2015).

### Cell cluster identification and cell type–specific TEs

We then used the DGE matrix to filter out cells with low complexity transcriptome or potential cell duplets based on total number of transcripts and genes, as described by Farrell et al. (2018). TE expression (including both gene-dependent and self-expressed) is ∼8% of the total transcriptome, so we increased the threshold for maximum reads and genes by 8% for each developmental stage. Our thresholds for gene and unique molecular identifiers (UMIs) for each development stage were as follows: high stage (1000–8100 genes, 1500–43,200 UMIs), oblong stage (625–8100 genes, 1500–32,400 UMIs), dome stage (800–4104 genes, 2000–21,600 UMIs), 30% epiboly (625–3240 genes, 1000–18,900 UMIs), 50% epiboly (600–4320 genes, 1500–27,000 UMIs), shield (600–2700 genes, 1000–16,200 UMIs), 60% epiboly (600–3780 genes, 1500–24,300 UMIs), 75% epiboly (600–3456 genes, 1400–21,600 UMIs), 90% epiboly (500–3780 genes, 1000–21,600 UMIs), bud stage (500–3456 genes, 100–18,900 UMIs), three-somite stage (500–3240 genes, 1000–13,500 UMIs), and six-somite stage (500–3240 genes, 1000–13,500 UMIs). We also excluded cells with unusually high mitochondria content (>45% of total reads per cell), an indication of stressed cells or cell apoptosis. After filtering, we had 45,127 cells for downstream analysis. We then used Seurat v3.0 (Stuart et al. 2019) to correct for batch effects and identified cell clusters based on expression of both genes and TE families (*dims = 143*, *resolution = 5.8*). We then identified cell type–specific TEs based using FindAllMarkers (*min.pct = 0.2*, *logfc.threshold = 0.25*, *min.diff.pct = 0.2*, *only.pos = TRUE*, *return.thresh = 0.05*).

### Pseudotime tree of single-cell TE expression

To obtain the cell trajectory across the developmental stages, we constructed a pseudotime tree based on both gene and TE family expression. We used the R package *URD* to conduct a diffusion map and flood stimulation (n = 1500) for all cells, as described by Farrell et al. (2018). We then defined the root as cells at the high stage and tip clusters from the six-somite stage using Infomap-Jaccard clustering. We simulated 10,000 random walks for all cells between root and tip clusters and reconstructed a cell trajectory tree from the simulation results. We then used a force-directed layout to visualize the reconstructed tree.

### In situ hybridization

To validate the TE expression from our single-cell analysis, we performed in situ hybridization in zebrafish embryos as described previously (Thisse and Thisse 2008). We amplified probes for ERV-2 LTR by using primers 5′-ACATNCCAGCTAGGAGGGACATT-3′ and 5′-CCTTTATTGAGACGTGTTGGTTAATCTGCAGT-3′, *pol* region of ERV1-3 by 5′- GATCCACAAACAGGCCAGAA-3′ and 5′-ACCTGCACACAAACATCGGA-3′, and *foxc1a* by 5′-CAGTCTTCTTGACGACTGTTCTTC-3′ and 5′-TAATCGAAATACTGGTTTGGTC-3′ from wild-type TU embryos, and then cloned them into pMiniT 2.0 for in vitro transcription. The mRNAs of ERV2-LTR, ERV1-3-I, and *foxc1a* were used as probes to hybridize embryos collected at 6.75 and 11 hpf, respectively. The RNA was labeled by DIG color and imaged by a ZEISS stereo microscope.

### Software availability

Scripts and analyses for genomic analyses are available at GitHub (https://github.com/vaquerizaslab/Chang_et_al_Zebrafish_TEs) and as Supplemental Code.

## Supplementary Material

Supplemental Material
