# Supplementary material for: Zebrafish transposable elements show extensive diversification in age, genomic distribution, and developmental expression
Source: Genome Res. 2022 Jul;32(7):1408–23. doi: 10.1101/gr.275655.121 (PMC9341512; doi:10.1101/gr.275655.121)
Supplement: Supplemental Material [file supp_32_7_1408__DC1.html]

Zebrafish transposable elements show extensive diversification in age, genomic distribution, and developmental expression — Supplemental Material 

# Zebrafish transposable elements show extensive diversification in age, genomic distribution, and developmental expression

## Supplemental Material

- Supplementary\_Code.tar.zip
- Supplementary\_material.docx
- Supplementary\_data\_1.tar.gz
- Supplementary\_data\_2.tar.gz
- Supplementary\_data\_3.tar.gz
- Supplementary\_data\_4.zip
- Supplementary\_data\_5.tar.gz
- Supplementary\_data\_6.tar.gz
