# Supplementary material for: Zebrafish transposable elements show extensive diversification in age, genomic distribution, and developmental expression
Source: Genome Res. 2022 Jul;32(7):1408–23. doi: 10.1101/gr.275655.121 (PMC9341512; doi:10.1101/gr.275655.121)
Supplement: Supplemental Material [file supp_gr.275655.121_Supplementary_material.docx]

**Supplementary Material**

**Zebrafish transposable elements show extensive diversification in age, genomic distribution, and developmental expression**

Ni-Chen Chang^1,*^, Quirze Rovira^2,*^, Jonathan Wells^1,*^, Cédric Feschotte^1,#^, Juan M. Vaquerizas^2,3,4,#^

1. Department of Molecular Biology and Genetics, Cornell University, Ithaca, New York 14850, USA.
2. Max Planck Institute for Molecular Biomedicine, Roentgenstrasse 20, 48149 Muenster, Germany.
3. MRC London Institute of Medical Sciences, Du Cane Road, London W12 0NN, UK.
4. Institute of Clinical Sciences, Faculty of Medicine, Imperial College London, Du Cane Road, London W12 0NN, UK.

* These authors contributed equally

# Correspondence to: C.F. ([cf458@cornell.edu](mailto:cf458@cornell.edu)), or J.M.V. ([j.vaquerizas@lms.mrc.ac.uk](file:///Users/jonwells/Downloads/j.vaquerizas@lms.mrc.ac.uk))

**Table of contents**

1. Supplementary Figures
2. Supplementary Data
3. Supplementary Code

**Supplementary Figures**

***Supplementary Figure 1. Correlation between mean divergence from consensus and median terminal branch length.***

*This analysis serves to validate the use of terminal branch lengths as a measure of age. It correlates strongly with the more widely used metric, “divergence from consensus sequence”, and is less sensitive to inflation as a result of TE family substructure. This can be seen in the case of LTR elements, which frequently have independently active subfamilies, and for which the divergence from consensus measure increases more rapidly than median terminal branch length.*

******

***Supplementary Figure 2. TE length correlates with age.***

*A. Differences in consensus sequence lengths between classes. Wilcoxon rank-sum test P-values given above boxes. B. Correlation between consensus sequence length and age for different TE classes. Values for r are calculated with Spearman’s rank correlation test.*


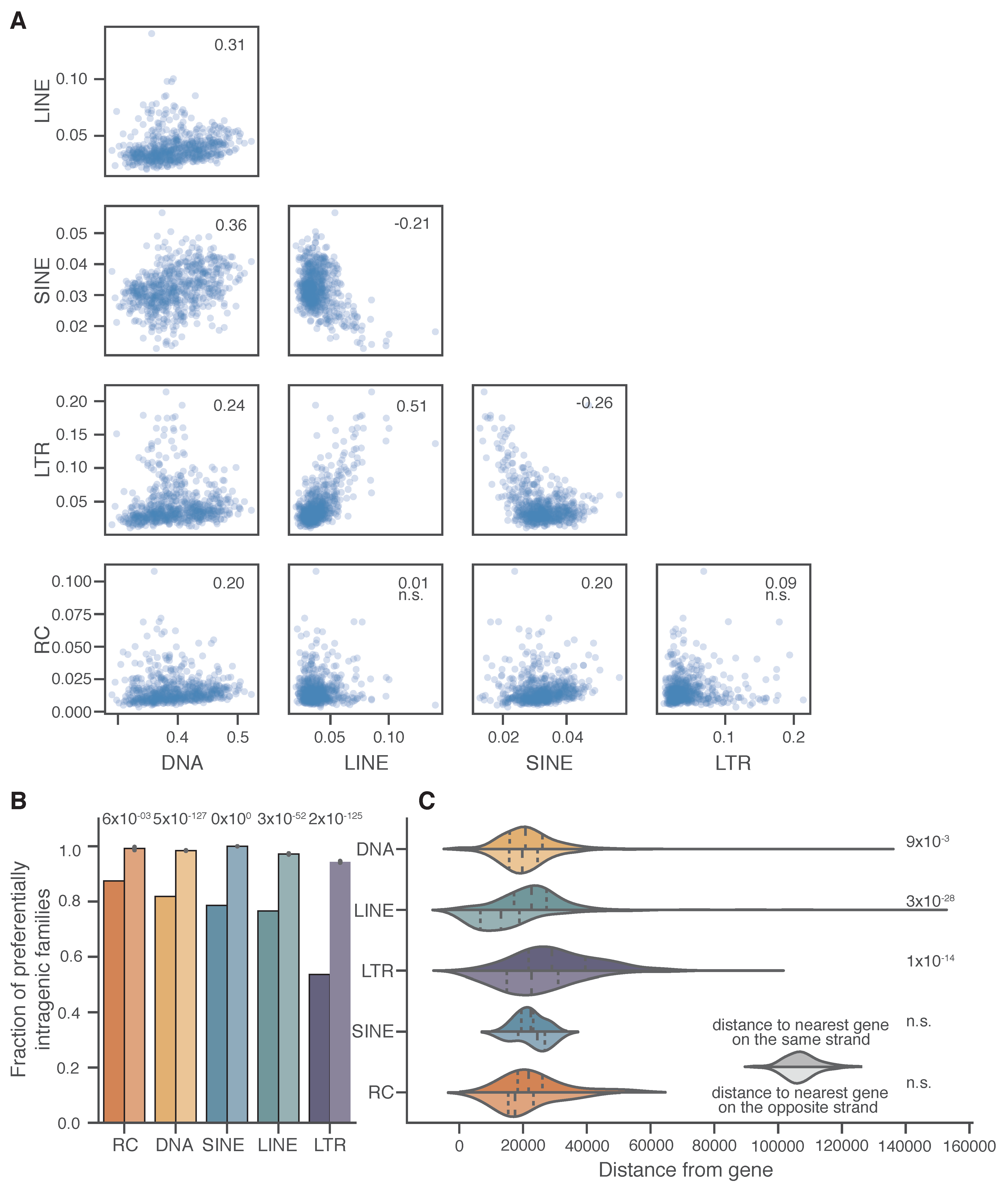
 ***Supplementary Figure 3. Replicate of Figure 2 with Chromosome four omitted from analysis.***

*Panels A, B and C correspond to panels C, D and E of Fig. 2 in the main body of text.*


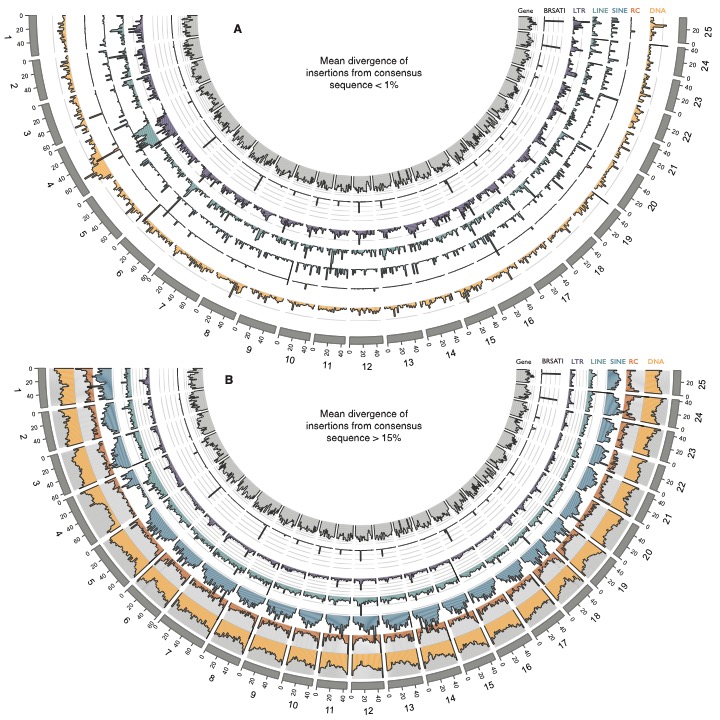


***Supplementary Figure 4. Genomic distribution of old and young insertions***

*Relates to figure 2A. A. Young insertions (<2% diverged from the TE family consensus sequence) are enriched in pericentromeric regions and on the long arm of Chromosome 4. This is consistent with TE families preferentially targeting repetitive or heterochromatic regions. B. When restricting the analysis to older insertions (>15% diverged from consensus), the patterns are broadly similar, with the exception of Chromosome 4, which is depleted of TEs, consistent with increased turnover of insertions.*


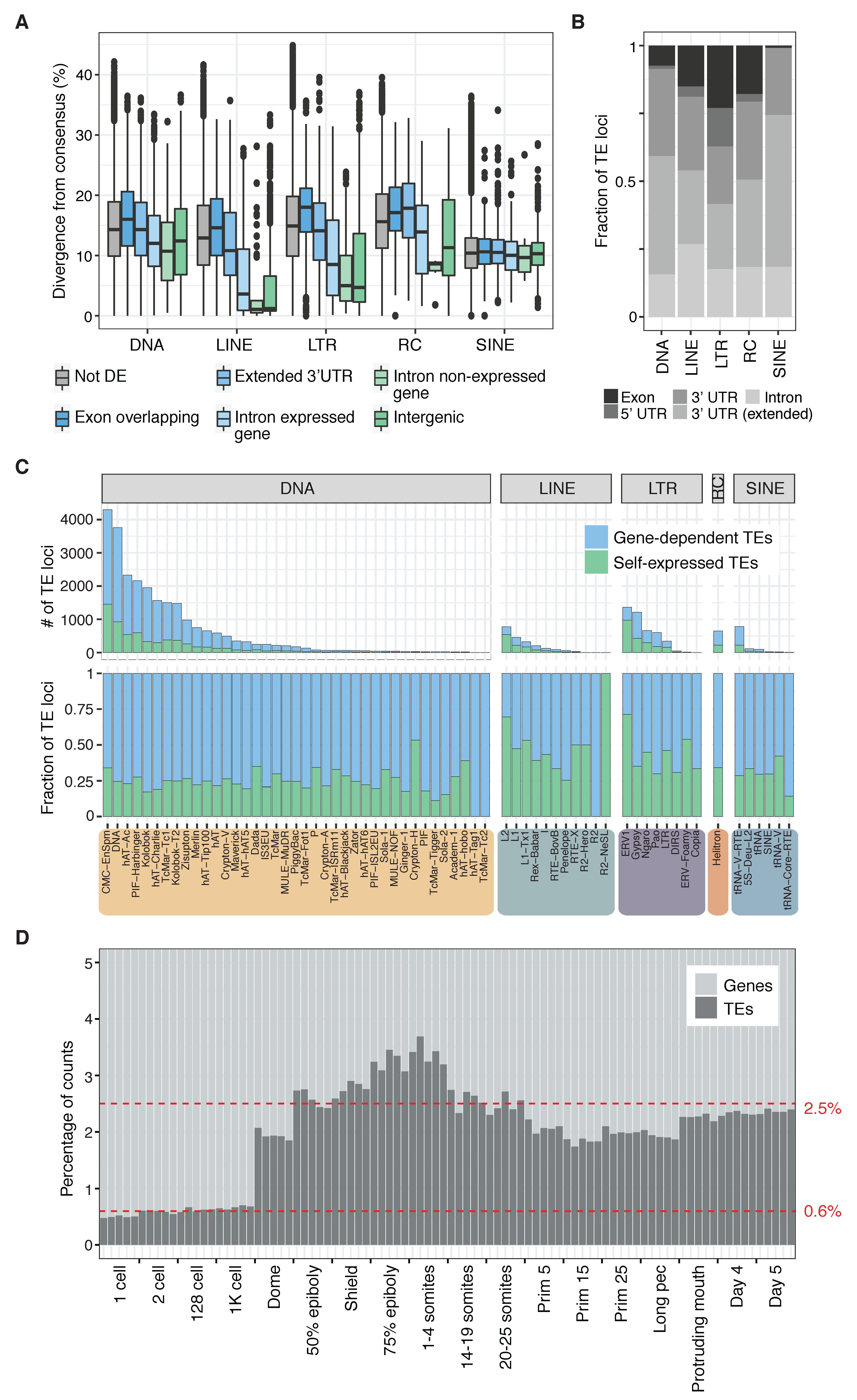


***Supplementary Figure 5.***

*A. Boxplot of divergence from consensus sequence for differentially expressed self-expressed TEs from all TE categories. B. Fraction of differentially expressed gene-dependent TE loci contained in different gene components. C. Number (top) and fraction (bottom) of differentially expressed TE loci in gene-dependent or self-expressed categories split by TE family and TE class. D. Percentage of read counts assigned to genes or TEs for each sample. Red dashed line marks the mean of TE assigned reads for pre-ZGA (lower) and post-ZGA (upper) samples.*

******

***Supplementary Figure 6. Transcription start sites are enriched within differentially expressed, self-expressed loci.***

*We defined transcription start sites as peaks of CAGE-seq that were completely contained within the boundaries of a given TE locus, and counted the peak numbers for three categories of TE loci: 1. gene-dependent, 2. self-expressed and differentially expressed, and 3. self-expressed and differentially expressed only in clusters B and C. P-values calculated with Fisher’s exact test, comparing self-expressed vs gene-dependent categories.*


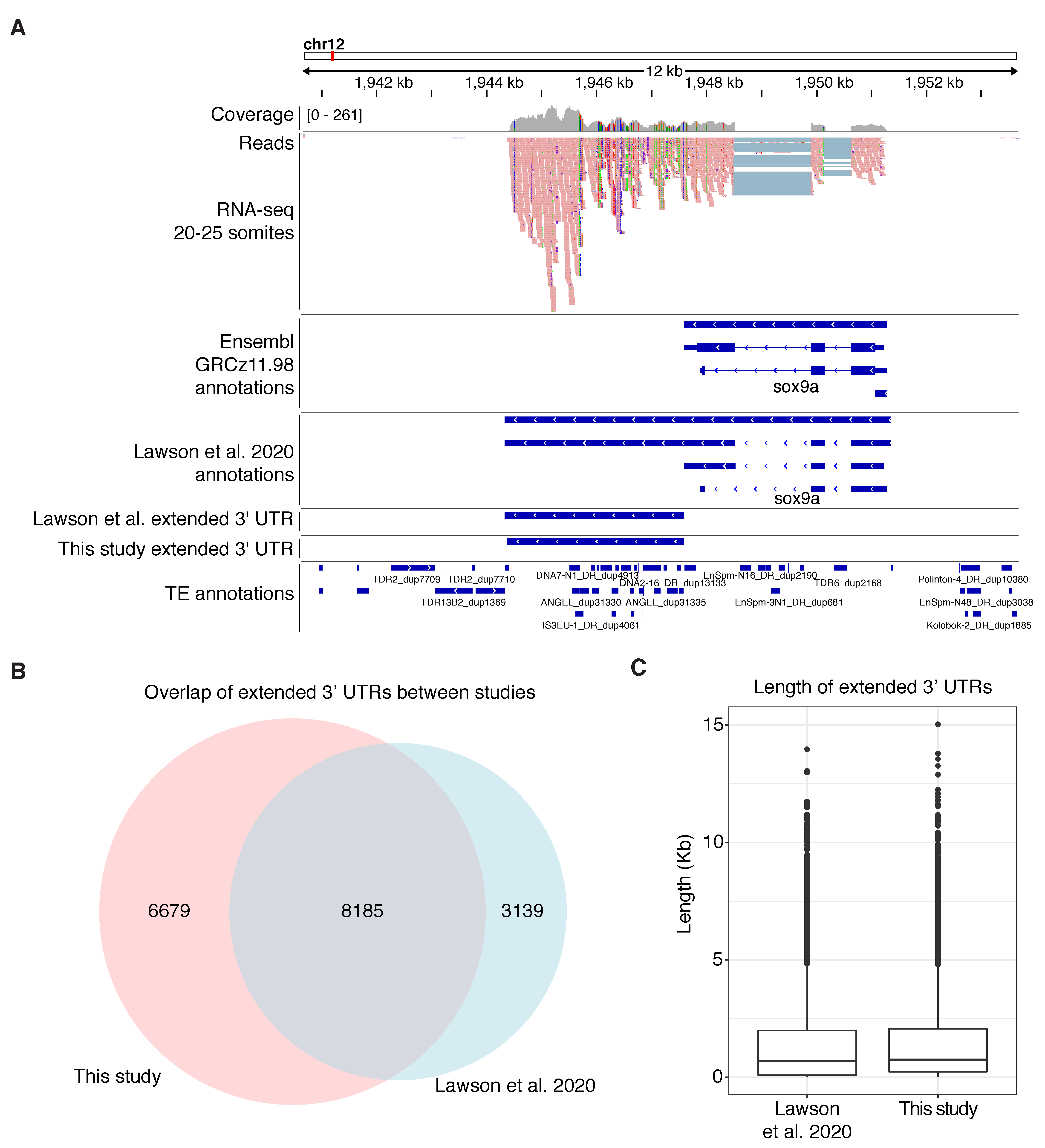


***Supplementary Figure 7.***

*A. Genome browser snapshot of a representative locus showing un-annotated 3’ UTR region on gene sox9a. RNA-seq track shows merged replicates at 20-25 somites. Ensembl GRCz11.98 and Lawson et al. 2020 annotations are shown, together with extracted extended 3’ UTR region from Lawson et al. and this study. In this study, transcriptome assembly using StringTie was used to find extended 3’ UTR regions using White et al. 2017 RNA-seq data. B. Overlap of extended 3’ UTR regions between Lawson et al. and this study. C. Length of extended 3’ UTR regions for Lawson et al. 2020 and this study show similar distribution.*
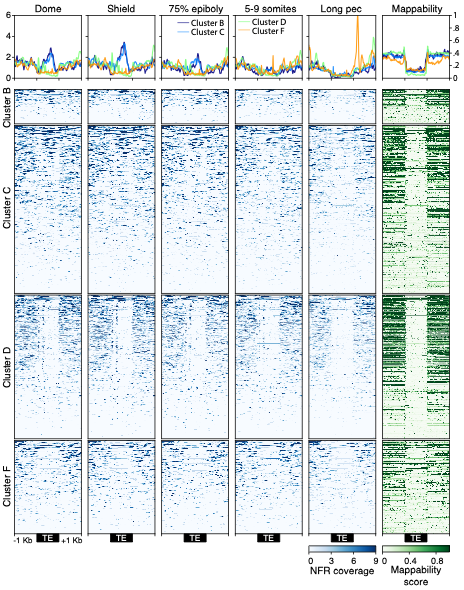


***Supplementary Figure 8. ATAC-seq signal on ERV1 TE family during early development.***

*ATAC-seq data* (Marlétaz et al. 2018)*was analysed using Genrich (see methods). Coverage over nucleosome free regions is shown for loci of the ERV1 TE family that are both self- and differentially expressed, split by cluster as in Figure 3D. The top row shows the average signal across each cluster. The column on the right depicts mappability scores calculated using the GEM library tool* (Derrien et al. 2012) *for a kmer length of 50bp. ERV1 loci in cluster B and C show an increased chromatin openness signal in the TE body on the development stages in which they are active.*

***Supplementary Figure 9.*** ***Nanog-like ChIP-seq analysis.***

*A. The heatmap shows a binding profile of nanog-like transcription factor on ERV1-N6 (61 out of 188 loci). B. Screenshots of peaks of Nanog-like binding sites on two ERV1-N6 elements. C. The Nanog-like motifs are located within LTRs of ERV1-N6 and also upstream the TATA box and TSS site.*

***
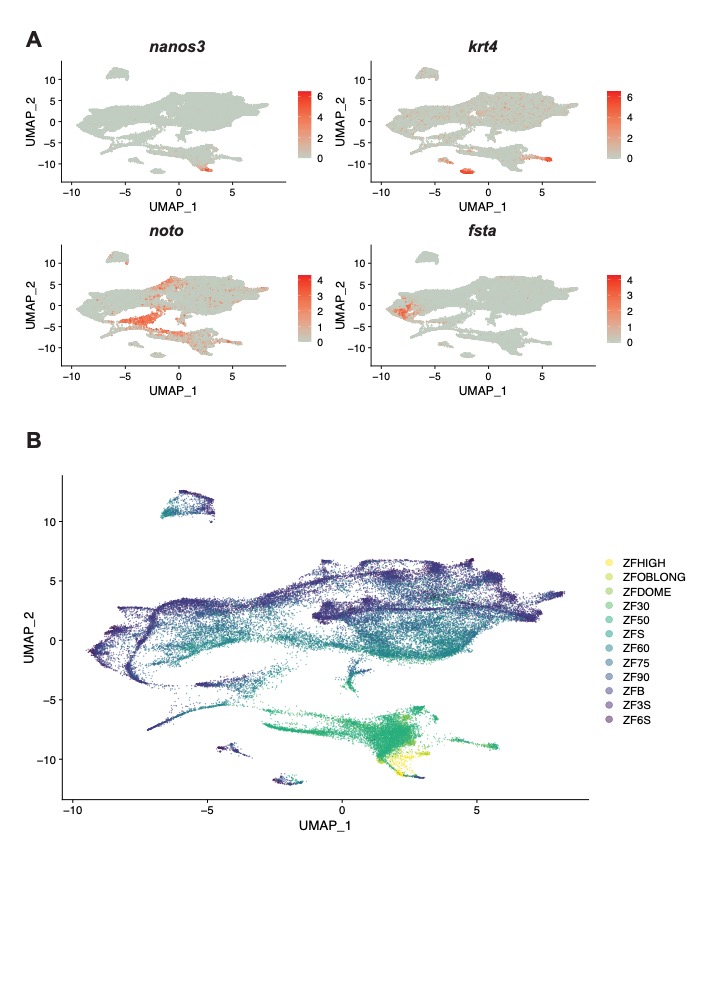
***

***Supplementary Figure 10.***

*Gene markers and development stages mark the cell lineages in UMAP from single cell sequencing. A. UMAP shows the cell lineage marked by nano3, the primordial germ cell lineage; krt4, the EVL cell lineage; noto, the notochord lineage; fsta, the cephalic mesoderm lineage. B. Cells cluster together with its closet developmental stages.*

***Supplementary Figure 11.***

*Reads mapped to individual loci of A. BHIKHARI and B. ERV1-3 in Bud stage from single-cell RNA-seq. From the 98 self-expressed loci of BHIKHARI, 97 of them are expressed. Of 45 self-expressed loci of ERV1-3, 43 are expressed, but most reads are from the 10 highly expressed loci.*

**Supplementary Data**

***Supplementary Data 1.*** *Primary RepeatMasker output file and summary table, along with processed data generated from ParseRM.pl (see methods). All downstream analyses and RNA-seq mapping were performed using these TE annotations.*

***Supplementary Data 2.*** *Multiple sequence alignments and corresponding phylogenetic trees of all families with at least 10 sequences of at least 100bp in length. See methods for construction details.*

***Supplementary Data 3.*** *Summary table containing genomic attributes of all TE families analyzed in this project. A README file with column descriptions is included within.*

***Supplementary Data 4.*** *GTF file with TE annotations. TE classification and differential expression cluster information included in the attribute’s columns.*

***Supplementary Data 5.*** *Count tables from Telescope with number of raw counts or normalized counts assigned to each TE.*

***Supplementary Data 6.*** *Processed data from scRNA-seq analysis. Included files are raw counts for each cell, UMAP coordinated and cell markers for each cell cluster.*

**Supplementary Code**

Scripts and analyses for genomic analyses are available as Supplemental Code, which and at GitHub: <https://github.com/vaquerizaslab/Chang_et_al_Zebrafish_TEs>.
